# CD39 and LDHA affects the prognostic role of NLR in metastatic melanoma patients treated with immunotherapy

**DOI:** 10.1186/s12967-023-04419-6

**Published:** 2023-09-08

**Authors:** Domenico Mallardo, Mario Fordellone, Andrew White, Margaret Ottaviano, Francesca Sparano, Michael Bailey, Arianna Bianca Facchini, Sufey Ong, Piera Maiolino, Corrado Caracò, Sarah Church, Ernesta Cavalcanti, Sarah Warren, Alfredo Budillon, Alessandra Cesano, Ester Simeone, Paolo Chiodini, Paolo Antonio Ascierto

**Affiliations:** 1 Istituto Nazionale Tumori IRCCS Fondazione G. Pascale, Naples, Italy; 2 Universitiy of Campania “Luigi Vanvitelli”, 81100 Naples, Italy; 3 NanoString Technologies Inc, Seattle, WA USA

**Keywords:** Melanoma, NLR, CD39, HDLA, TGFβ, Biomarker, Gene signature, Transcriptomic analysis, Brain metastases

## Abstract

**Background:**

Identifying response markers is highly needed to guide the treatment strategy in patients with metastatic melanoma.

**Methods:**

A retrospective study was carried out in patients with unresectable/metastatic melanoma (stage IIIb–IV), treated with anti-PD-1 in the first line setting, to better explore the role and the timing of neutrophil/lymphocyte ratio (NLR) as potential biomarker of response. The relationship of NLR with inflammation-immune mediators and the underlying negative effect of raising NLR during immunotherapy, have been investigated with transcriptomic gene analysis.

**Results:**

The results confirmed previous findings that a high baseline NLR is associated with a poorer prognosis and with higher serum level of lactate dehydrogenase (LDH), regardless of the presence of brain metastases. The transcriptomic analysis showed that high baseline NLR is associated with a characteristic gene signature *CCNA1*, *LDHA* and *IL18R1*, which correlates with inflammation and tumorigenesis. Conversely, low baseline NLR is associated with the signature *CD3*, *SH2D1A*, *ZAP70* and CD45RA, linked to the immune-activation. The genes positively associated with NLR (*CD39* (*ENTPD1*), *PTEN*, *MYD88*, *MMP9* and *LDH*) are involved in processes of immunosuppression, inflammation and tumor-promoting activity. Increased expression of *CD39* correlated with TGFβ_2_, a marker of the N2 neutrophils with immunosuppressive activity.

**Conclusions:**

These results suggest that increasing NLR is associated with an increased neutrophil population, with polarization to the N2 phenotype, and this process may be the basis for the negatively prognostic role of NLR.

**Supplementary Information:**

The online version contains supplementary material available at 10.1186/s12967-023-04419-6.

## Background

The improvement of survival outcomes in patients with advanced melanoma due to the introduction of immune checkpoint inhibitors (ICIs) and targeted therapy (TT) for BRAF mutated melanoma, is undoubtedly very remarkable; nevertheless a proportion of patients still have poor prognosis [1–3]. Therefore, a growing line of research is focusing on the identification of potential biomarkers that may guide the treatment strategy [4–6].

Immunosuppression in the tumor microenvironment, induced by systemic and chronic inflammation and mediated by several types of circulating cells, is a known factor that favors tumor growth and cancer cell migration [7]. Several parameters of immune activity and inflammation have been investigated as candidate markers for prognosis or treatment effect; in this context, some studies have demonstrated that an elevated neutrophil-to-lymphocyte ratio (NLR) predicts poor outcomes in patients with solid cancers [8]. More recently, elevated NLR, as well as elevated derived NLR that is calculated from absolute neutrophil count (ANC) and white cell count, were found to be independent predictors of reduced survival and increased risk of progression in melanoma patients receiving ipilimumab or nivolumab [9, 10]. Conversely, patients with metastatic melanoma, who developed immune-related adverse events while treated with ICIs, had an increased response rate if the NLR was elevated [11]. Overall, current evidences suggest that NLR may be used to predict response to immunotherapy in melanoma patients, although its timing may be further investigated. Additionally, in patients with advanced *BRAF* wild-type melanoma, the concomitance of basal elevated lactate dehydrogenase (LDH) and NLR increasing on treatment with ICIs has been associated with reduced progression-free survival (PFS) [12]. This result confirmed previous reports of LDH as a negative prognostic marker in immunotherapy for melanoma [13, 14].

With the aim to better explore the relationship of NLR with inflammation-immune mediators and the underlying negative effect of raising NLR during immunotherapy, we carried on a transcriptomic gene analysis of peripheral blood mononuclear cells (PBMC) from patients with metastatic melanoma treated with anti-PD1 agent.

## Patients and methods

### Patients

A retrospective study was carried out at Istituto Nazionale Tumori—IRCCS—Fondazione “G. Pascale,” Naples, Italy. The study was approved by the Ethics Committee of Istituto Nazionale Tumori—IRCCS—Fondazione “G. Pascale”, Naples, Italy, protocol number 17/17 oss. The study was performed in accordance with the revised version of the declaration of Helsinki (52nd WMA General Assembly, Edinburgh, Scotland, October 2000).

Consecutive adult patients with histologically confirmed unresectable/metastatic melanoma (stage IIIb–IV according to American Joint Committee on Cancer AJCC 7th Edition), treated with anti-PD-1 agent in the first line setting between April 2016 and June 2018, were included in the analysis. All patients provided their written informed consent.

### Methods

#### Survival outcomes measures

RECIST 1.1 criteria were used to radiologically evaluate the tumor response as complete response (CR), partial response (PR), stable disease (SD), or progressive disease (PD). The following parameters were recorded: response rate (RR) at first assessment; progression free survival (PFS)—calculated from the time of the first dose of anti-PD-1 agent to radiological progression, death or lost to follow-up, whichever occurred first; overall survival (OS) calculated from the time of the first dose of anti-PD1 agent to death or lost-to-follow-up, whichever occurred first; disease control rate (DCR) defined as the sum of CR, PR, and SD > 1 year); objective response rate (ORR) defined as the sum of CR and PR). Response was evaluated based on DCR: patients with SD < 1 year were classified as non-responders, patients with SD ≥ 1 year were classified as responders.

### Bio-umoral analysis

LDH serum level and NLR were assessed at baseline; NLR was recorded after 3 months of treatment with anti-PD-1 ICI. Blood samples from enrolled patients were collected at baseline to conduct a gene profile analysis. RNA from PBMCs was extracted using RNA blood mini-Kit (Qiagen). Purified RNA was used for hybridization and underwent to gene profiling analysis on NanoString nCounter through PanCancer IO 360™ panel, characterized by human genes associated with immune activation, inflammation and control of the cell cycle. Gene data were normalized using nSolver Version 4.0 Software; NanoString. Counts were normalized to External RNA Controls Consortium (ERCC) technical controls and 30 housekeeping genes.

### Statistical analysis

Continuous variables were reported as either the means and standard deviation or median and interquartile ranges (IQRs) according to their distribution, as assessed by the Shapiro–Wilk normality test. Categorical variables were reported as percentages. Differences in characteristics of patients between the groups of low and high NLR were tested by t-test or Wilcoxon test (according to their distribution) and Pearson chi-squared test for continuous and categorical variables, respectively. To measure the linear association between continuous variables, the Pearson correlation coefficient was used if variables had a normal distribution; otherwise, the Spearman’s correlation coefficient was calculated.

PFS and OS were outcome survival measures to assess differences in prognosis according to groups of low and high NLR. The optimal cut-point to define the low and high NLR subgroups was selected through the log-rank test maximization performed by a cross-validation approach. The differences in prognosis between the low NLR group and high NLR group were tested by log-rank test and represented by Kaplan–Meier curves. The inverse Kaplan–Meier approach estimated median follow-up.

The discriminant analysis for sparse data performed via partial least squares procedure (sPLS-DA) was applied to identify gene signatures associated with the NLR groups. The sparse variant of PLS-DA enables the selection of the most predictive or discriminative features in the data to classify the samples [15]. sPLS-DA performs variable selection and classification in a one-step procedure, where the lasso penalization applies only to the loading vector associated with the X data set. In particular, sPLS-DA is a versatile algorithm that can be used for predictive and descriptive modeling and discriminative variable selection when the matrix of predictors has more variables than observations and when there is multicollinearity among variables. The principal outputs of this statistical model consist in *(i)* a set of latent scores (i.e., components or signature score) that are defined as a linear combination of the original variables projected in a new subspace and in *(ii)* a loadings matrix to define the relationships among the variables and the components.

To select the optimal number of sPLS-components, a cross-validation approach was performed to maximize the area under curve (AUC) of the Receiver Operating Characteristic (ROC). Finally, to validate the selection of the genes identified through the maximum relationship with the sPLS-components, a Principal Component Analysis (PCA) on this gene set was performed. In particular, the genes with the lowest explained variance on the first PCA component were discarded.

## Results

Overall, 78 patients were included in the analysis. Demographic and baseline clinical data are reported in Table 1. Briefly, 37 patients (53%) were males, the median age was 61 years (range, 27–91 years), 19 (24%) patients had brain metastases, 59 (76%) had BRAF wild-type melanoma and 16 (21%) had BRAF mutations, while BRAF status was not known for 3 patients. Fifty-two patients (67%) received nivolumab, while the remaining 26 (33%) received Pembrolizumab.

**Table 1:** Patient characteristics

Characteristics	n = 78, n (%)
Age (years), median (range)	61 (27–91)
Gender:	
• Female	41 (53)
• Male	37 (47)
Melanoma AJCC VII stage:	
• Stage IV	74 (94)
• Stage IIIC	4 (5)
• Stage IIIB	1 (1)
Brain metastases at baseline	18 (23)
*BRAF* status:	
• Wild-type	59 (76)
• Mutation	16 (21)
• NA	3 (3)
M category:	
• M0	3 (4)
• M1a	11 (14)
• M1b	10 (13)
• M1c	54 (69)
LDH:	
• High	26 (33)
• Normal	34 (44)
• NA	18 (23)

The optimal cut-point to define the low and high NLR subgroups was 5.57 (Additional file 1: Figure S1). At baseline, the NLR was < 5.57 (low) in 66 (84.6%) patients and ≥ 5.57 (high) in 12 (15.4%), while the serum LDH level was normal in 34 (44%) patients and high in 26 (33%). There were no differences in age, gender, body mass index (BMI), *BRAF* mutation, M category, LDH level, and glycemia between patients with high and low NLR. Serum level of LDH was positively associated with NLR value at baseline (rho 0.268, 95% CI 0.0148–0.488, p = 0.0386).

Brain metastases were present at baseline in a higher proportion of patients with high NLR compared to those with low NLR [7 (58.3%) patients vs 12 (18.2%); p = 0.01].

### Response disease and survival outcomes

**Fig. 1:**
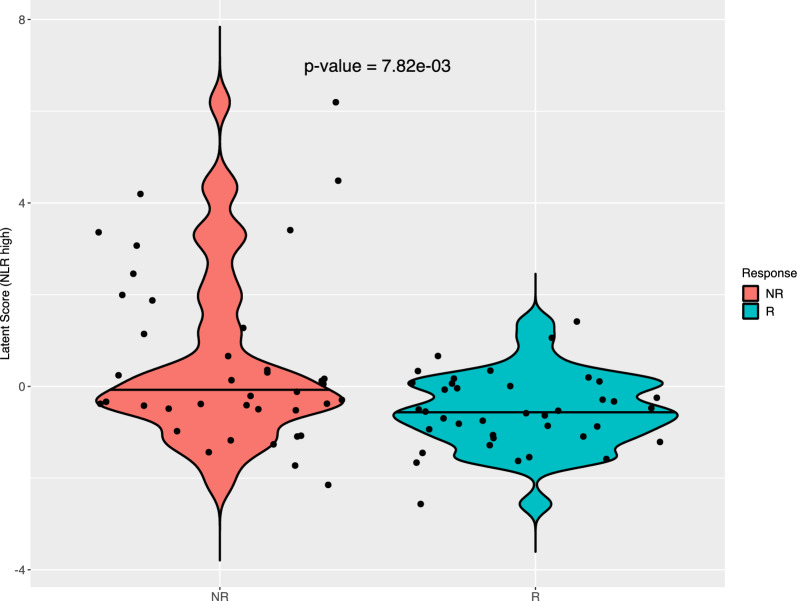
NLR according to response to treatment, at 3 months

**Fig. 2:**
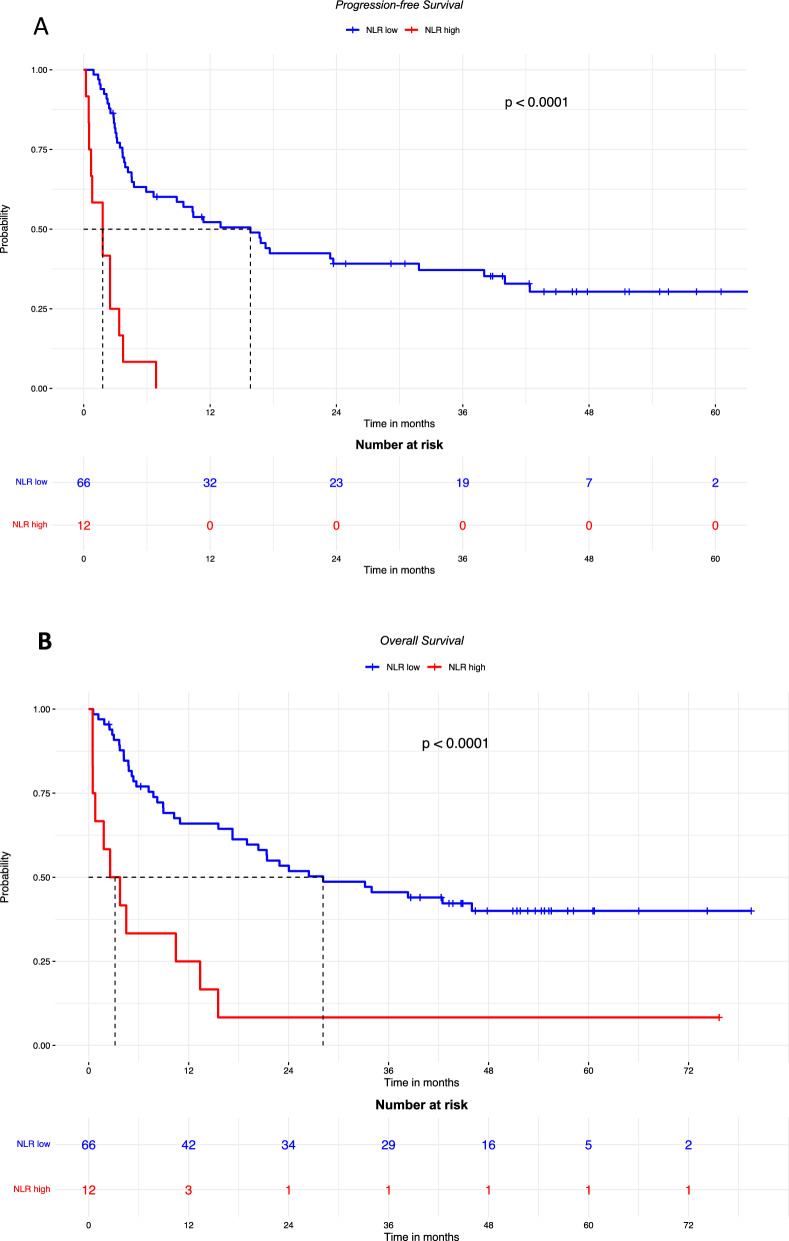
PFS (**A**) and OS (**B**) in patients with high or low baseline NLR. PFS: median follow-up was 54.7 months in patients with low NLR, and was not available for those with high NLR. OS: median follow-up was 51.8 months in patients with low NLR, and 75.7 months for those with high NLR

At the first assessment after 3 months of treatment, 9 (11%) patients achieved CR, 16 (21%) PR with an ORR of 32%, 17 (23%) SD with a DCR of 50% and 36 (46%) PD [Table 2]. Skin toxicity was recorded in 29 patients with low NLR (49.9%) and 1 with high NLR (8.3%) (p = 0.04), we did not observe any significant differences in other toxicities (Additional file 1: Table S1). High NLR was significantly more frequent in patients who had not responded to treatment (p = 0.00782) (Fig. 1). Patients with high basal NLR had shorter PFS and OS than those with low basal NLR with HR = 7.27 (95% CI 3.57–14.81; p < 0.0001) (Fig. 2A) and HR = 3.98 (95% CI 2.00–7.91) (Fig. 2B), respectively.

**Table 2:** Response to treatment at 3 months

Response to treatment	n = 78, n (%)
Response at first assessment:	
• Complete response	9 (11)
• Partial response	16 (21)
• Stable disease	17 (22)
• Progression disease	36 (46)
ORR	25 (32)
DCR	39 (50)

**Fig. 3:**
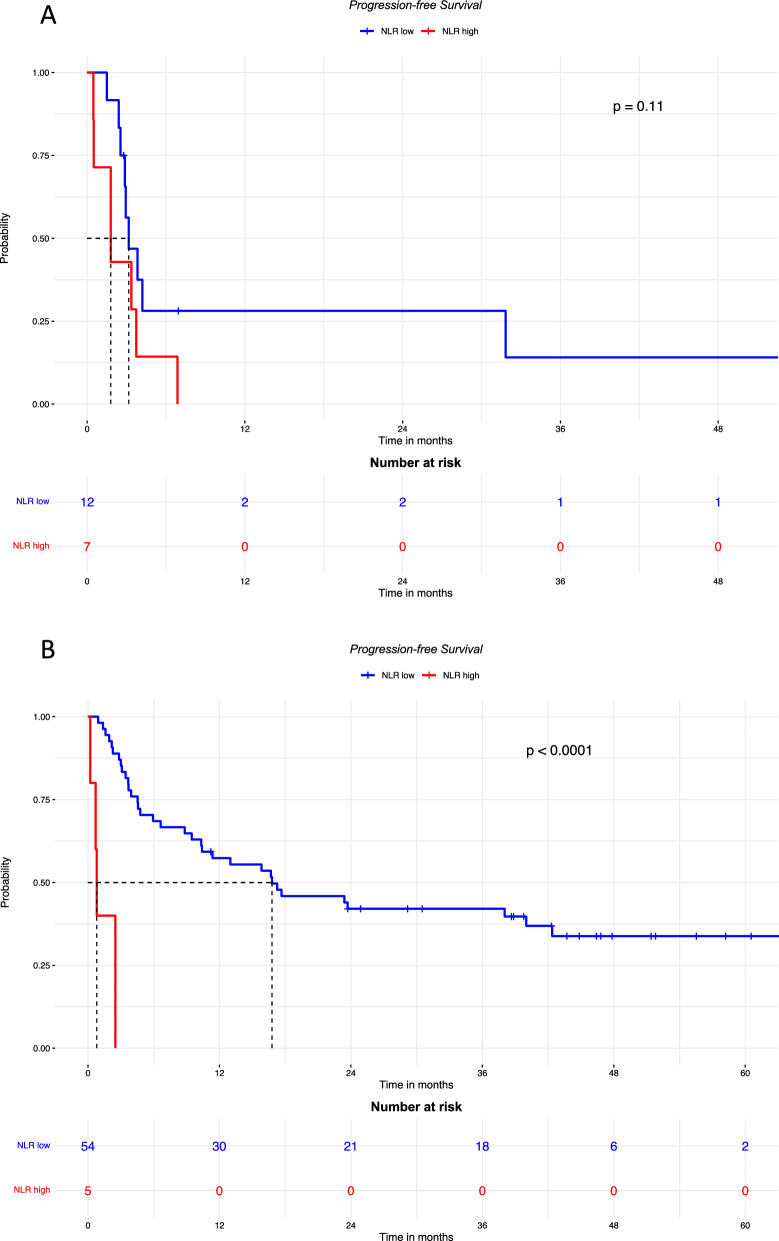

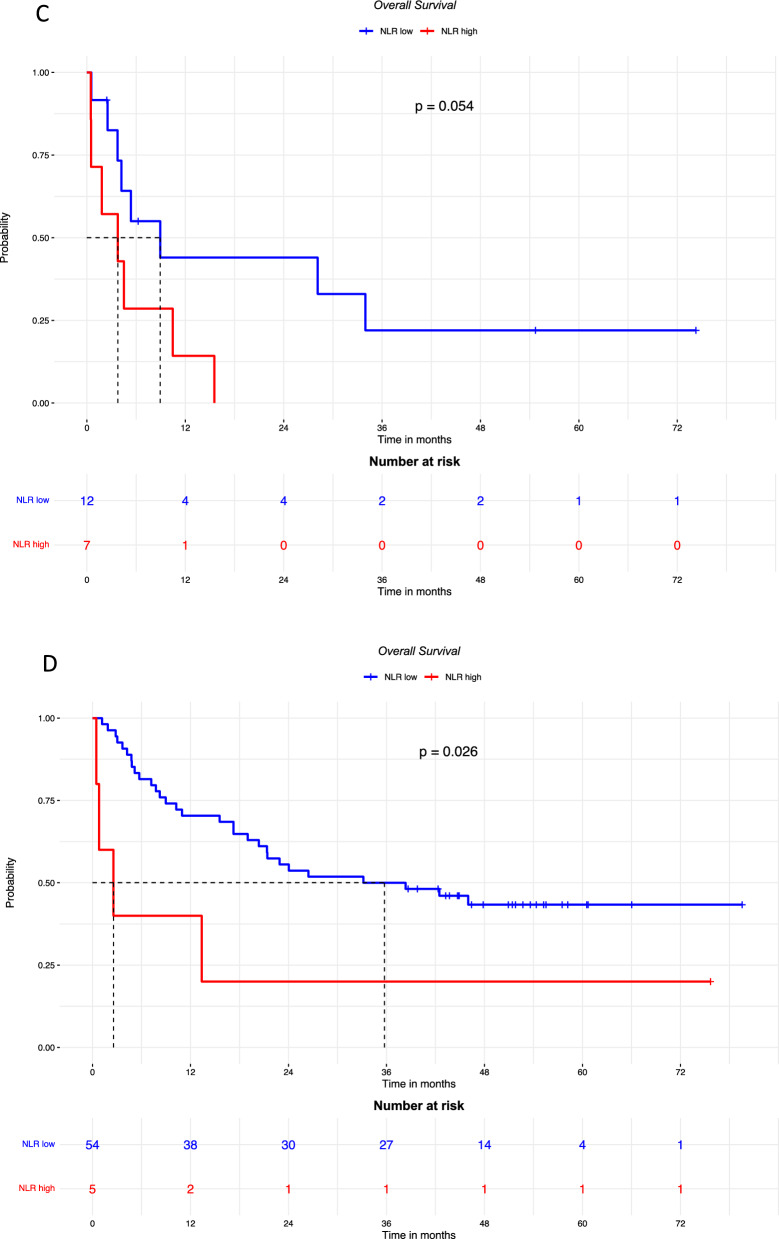
PFS in patients with brain metastases (**A**; median follow-up was 54.7 months in patients with low NLR, and was not available for those with high NLR) and without brain metastases (**B**; median follow-up was 44.8 months in patients with low NLR, and was not available for those with high NLR); OS in patients with brain metastases (**C**; median follow-up was 54.7 months in patients with low NLR, and was not available for those with high NLR) and without brain metastases (**D**; median follow-up was 51.8 months in patients with low NLR, and 75.7 months in those with high NLR), according to high or low baseline NLR

As high NLR is known to be associated with brain metastases present at baseline or subsequently [16, 17], we decided to rule out a possible role of brain metastases in the negative correlation of NLR with prognostic parameters, and the subgroups of patients with and without brain metastases were analyzed. As shown in Fig. 3, the PFS in patients with brain metastases was not significantly different according to NLR level and the HR for high NLR was 2.28 [95% CI 0.82–6.37 (p = 0.11)]. On the contrary, PFS was significantly poorer in patients without brain metastases if NLR was high compared to low NLR (HR 18.93 [95% CI 5.66–63.27] p < 0.0001).

The OS was poorer in the groups with high NLR both in patients with brain metastases (HR: 2.81 [95% CI 0.94–8.40] p = 0.054), and in those without brain metastases (HR 3.09 [95% CI 1.08–8.81] p = 0.026).

To evaluate the effect of an increased NLR after 3 months of treatment compared to baseline, the NLR_post_/NLR_baseline_ ratio was calculated and the optimal cut-point (i.e. 0.76) to define the subgroups of increased NLR vs no increase was identified (Additional file 1: Figure S2).

**Fig. 4:**
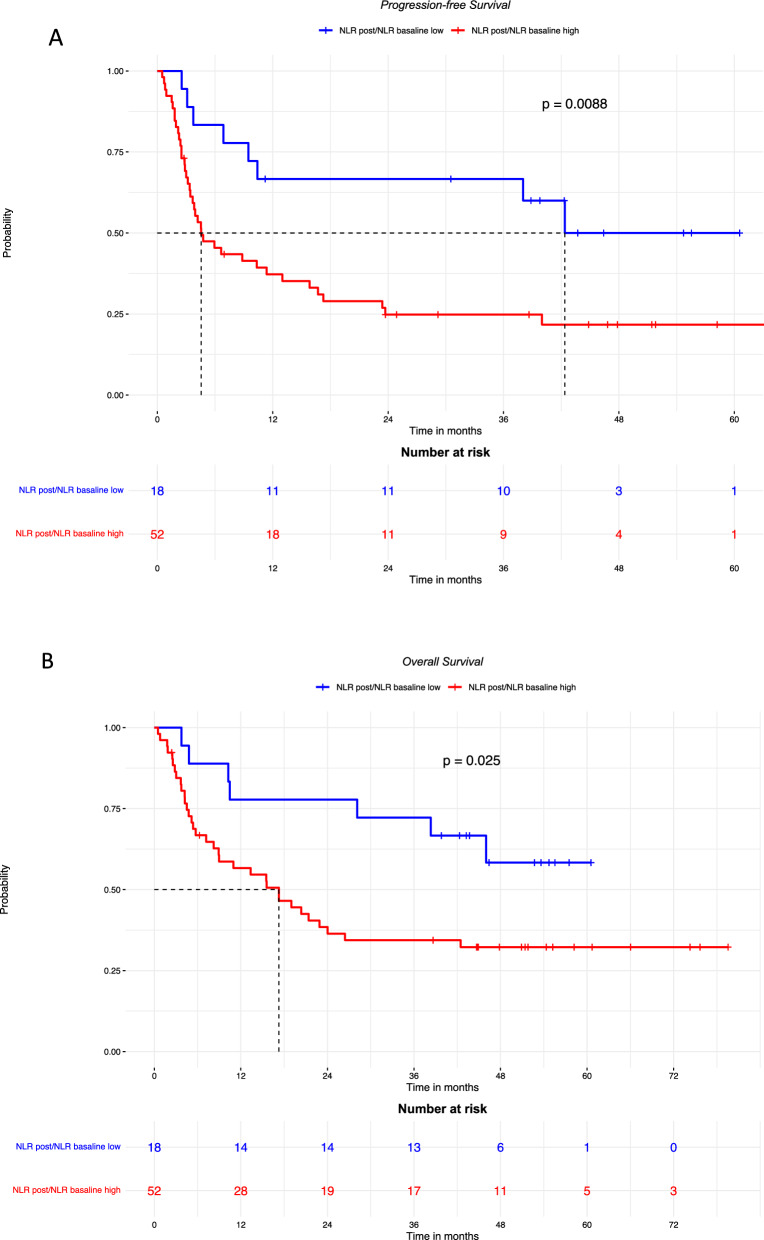
PFS (**A**; median follow-up was 43.7 months in patients with low NLR, and 46.8 months those with high NLR.) and OS (**B**; median follow-up was 52.7 months in patients with low NLR, and 51.8 months in those with high NLR.) according to rising or consistent LNR after 3 months of treatment

The patients who had a high NLR_post_/NLR_baseline_ (≥ 0.76) had poorer PFS and OS compared to those whose NLR did not increase after treatment (Fig. 4A, B).

### Transcriptomic analysis

**Fig. 5:**
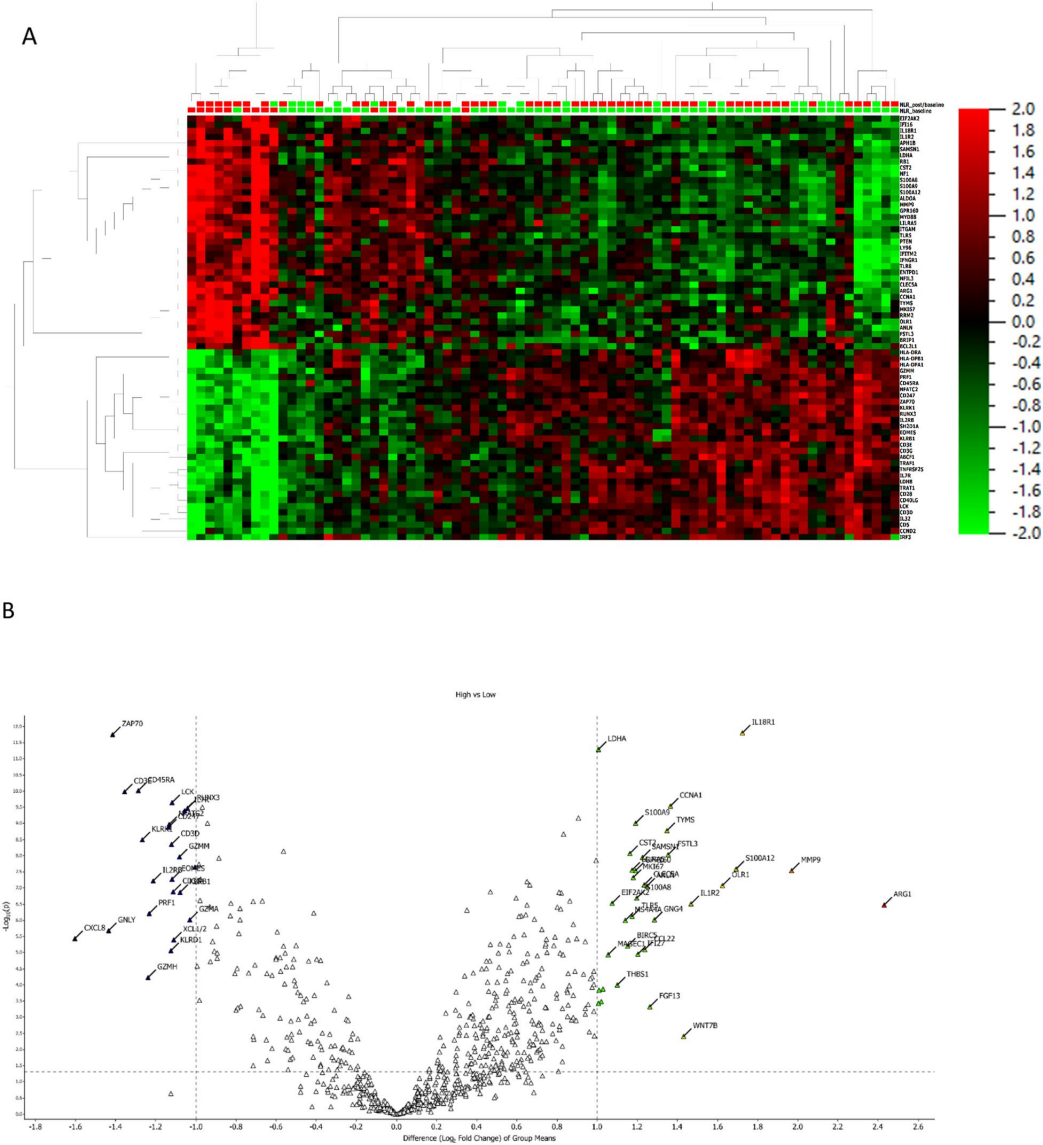
Transcriptomic analysis of PBMC obtained at baseline, according to NLR. **A** heat map representation. **B** Volcan plot; p values are reported on the Y axis; values reported over the orizontal dotted line are significant

**Fig. 6:**
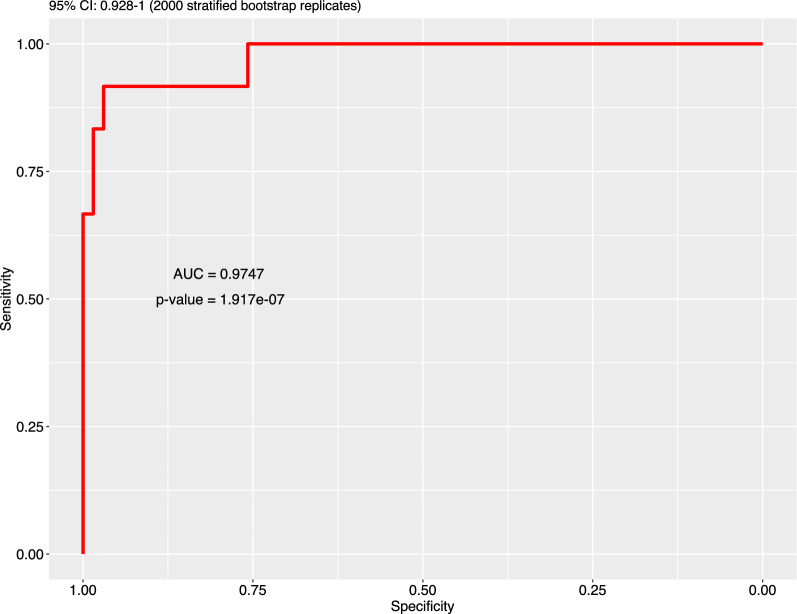
Accuracy of gene selection, through latent score, for baseline NLR

The transcriptomic analysis of PBMCs obtained at baseline identified a set of an optimized number of genes positively or negatively associated with NLR at baseline (Fig. 5 A, B; Additional file 1: Figures S3, S4, S5 and S6, Table S2) (Table 3). A gene signature was validated through a ROC curve (Fig. 6).

**Table 3:** Association of gene expression with NLR, at baseline

Gene	n	Rho	p-value
Positively associated with NLR			
* CD39* (*ENTPD1*)	78	0.663	< 0.0001
* PTEN*	78	0.034	< 0.0001
* MYD88*	78	0.662	< 0.0001
* MMP9*	78	0.749	< 0.0001
Negatively associated with NLR			
* HLA-DRA*	78	−0.473	< 0.0001
* HLA-DPB1*	78	−0.547	< 0.0001
* HLA-DPA1*	78	−0.558	< 0.0001
* CD5*	78	−0.557	< 0.0001
* CD28*	78	−0.578	< 0.0001
* NFATC2*	78	−0.660	< 0.0001
* CD247*	78	−0.610	< 0.0001
* ZAP70*	78	−0.723	< 0.0001
* IL2RB*	78	−0.499	< 0.0001
* CD3E*	78	−0.532	< 0.0001
* CD3G*	78	−0.558	< 0.0001
* IL7R*	78	−0.620	< 0.0001
* TRAT1*	78	−0.515	< 0.0001
* CD40LG*	78	−0.537	< 0.0001
* CD3D*	78	−0.525	< 0.0001
* IL32*	78	−0.511	< 0.0001

The gene signature *CD3*, *SH2D1A*, *ZAP70* and *CD45RA* was associated with a low baseline NLR, while a high baseline NLR was associated with *CCNA1*, *LDHA* and *IL18R1*.

*CD3E, SH2D1A, ZAP70*, and *CD45RA* were more represented at baseline in patients who responded to treatment at 3 months than in non-responders, while *CCNA1*, *LDHA* and *IL18R1* were more expressed by no-responders (Additional file 1: Figure S7).

In addition, NLR was positively associated with genes related to immunosuppression, inflammation and tumorigenesis: *CD39* (*ENTPD1*), *PTEN*, *MYD88*, *MMP9* and *LDH*. NLR was negatively associated with genes involved in the priming of immune activation: *HLA* genes, *CD28*, *CD5*, *CD247*, *NFATC2*, *ZAP70*, *IL2RB*, *CD3E*, *CD3G*, *CD3D*, *IL7R*, *TRAT1*, *CD40LG*, *IL32* (Table 3, Additional file 1: Table S3).

Increased expression of CD39 was associated with the markers of N2 polarization of neutrophils TGFβ_2_ (rho = 0.42; 95% CI 0.218–0.587; p = 0.0001) and TGFβR_1_ (rho = 0.541, 95% CI 0.362–0.681; p < 0.0001). It was inversely associated with expression of CD8A (rho -0.341, 95% CI −0.524 to −0.128; p = 0.0023), CD8B (rho −0.468, 95% CI −0.625 to -−0.274; p < 0.0001), CD4 (rho −0.356, 95% CI −0.536 to −0.145; p = 0.001), CD45RA (rho −0.619, 95% CI −0.739 to −0.459; p < 0.0001), and CD45RB (rho −0.247, 95% CI −0.445 to −0.0259; p = 0.0292).

**Fig. 7:**
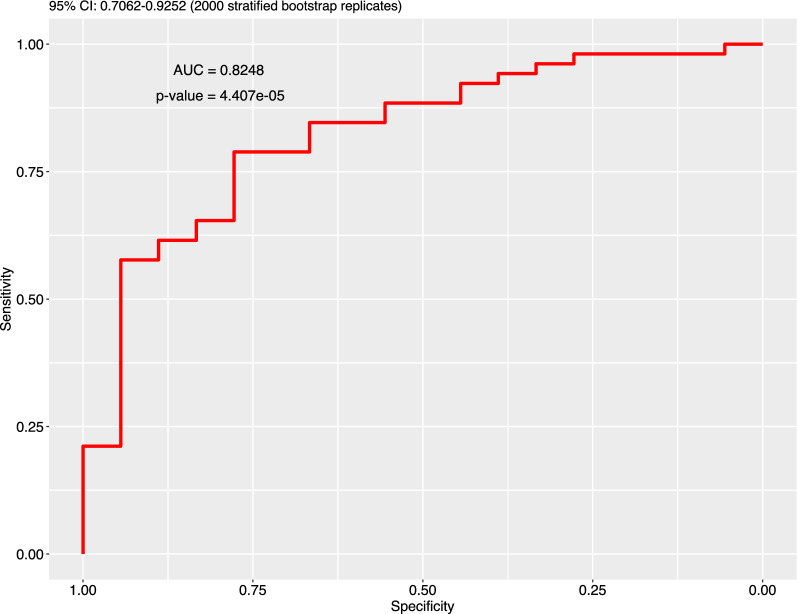
Accuracy of gene selection, through latent score, for high NLR_post_/NLR_baseline_

A gene signature including *IRF5* and *PPARGC1B* was associated with a high NLR_post_/NLR_baseline_ (Fig. 7, Additional file 1: Figures S8, S9).

While no clinical variable (age, gender, BMI, *BRAF* mutation, M category, LDH, brain metastases, glycemia) was associated with the NLR_post_/NLR_baseline_, the expression of *WNT5A* was positively associated (rho 0.298, 95% CI 0.067–0.498; p = 0.012, Spearman’s test) and the expression of *APNLR* was negatively associated (rho −0.313, 95% CI −0.511 to −0.084; p = 0.0083, Spearman’s test) with NLR_post_/NLR_baseline_.

## Discussion

This study investigated the relationship of NLR with the gene profiling of PBMC obtained from patients with metastatic melanoma who underwent immunotherapy with Anti-PD1 agent. The results confirmed previous findings that a high baseline NLR is associated with a poorer prognosis and correlates with high LDH serum level [9, 10, 12]. Although NLR has been associated with the presence of brain metastases [16, 17], we were not able to find a correlation between high NLR and the presence of brain metastases, conversely our results detected a NLR even higher in patients without brain metastases. Indeed, NLR impacts the PFS and OS of patients without brain metastases with a higher effect than those with such metastases. On the other hand, we did not observe any correlation between NLR and BMI, as previously observed in patients with Hodgkin lymphoma treated with an immune checkpoint inhibitor [18].

The transcriptomic analysis showed that patients with high NLR have the gene signature *CCNA1*, *LDHA* and *IL18R1*, which correlates with inflammation and tumorigenesis. The strong association with *LDHA* suggests that this enzyme isoform may be involved in a key mechanism for cancer progression. A low NLR at baseline was associated with the signature *CD3*, *SH2D1A*, *ZAP70* and *CD45RA.* These genes are correlated with the activation of immunity. Indeed, we have previously found that CD3 + CD45 + T-memory cells are positively correlated with the oncological outcomes of patients with stage IV melanoma treated with ipilimumab [19].

The genes positively associated with NLR [*CD39* (*ENTPD1*), *PTEN*, *MYD88*, *MMP9* and *LDH*] have immunosuppression, inflammation and tumor-promoting activity. On the contrary, the genes negatively associated with NLR (*HLA* genes, *CD28*, *CD5*, *CD247*, *NFATC2*, *ZAP70*, *IL2RB*, *CD3E*, *CD3G*, *CD3D*, *IL7R*, *TRAT1*, *CD40LG*, *IL32)* are involved in the priming of immune activation. We found that the increased expression of *CD39* was associated with TGFβ_2_ and TGFβR_1_. TGFβ is a marker of the N2 neutrophils with immunosuppressive activity [20]. *CD39* was also inversely associated with genes involved with memory cells and adaptive T cells, such as di CD8A, CD8B, CD4, CD45RA and CD45RB.

Based on these results, the role of CD39/ ENTPD1 in the negative effect of a high NLR on the oncologic outcomes of patients with advanced melanoma can be speculated. CD39 (*ENTPD1)* functions as the rate-limiting step in converting ATP to ADP [21]. Adenosine inhibits anti-tumor functions mediated by T cells and NK cells [22]. ENTPD1/CD39 is expressed in the tumor microenvironment, in vessels, B cells, NK cells, dendritic cells, monocytes, macrophages, regulatory T cells and monocyte-derived suppressor cells [23]. Upregulation of CD39 in the tumor microenvironment is associated with CD8 + T cell exhaustion signatures [24]. Additionally, T regulatory (Treg) cells may upregulate ENTPD1/CD39 in the tumor microenvironment resulting in immunosuppression and promotion of tumor growth [25].

These data suggest that a high NLR is shaped by an increased expression of CD39, resulting in activation of the adenosine pathway and an increased component of N2 neutrophils with reduced presence of lymphocytes in the tumor microenvironment. CD39 (ectonucleoside triphosphate diphosphohydrolase 1; encoded by ENTPD1) binds extracellular ATP (eATP) and converts it to extracellular adenosine mostly via a cascade involving the ecto-enzyme CD73 (also known as ecto-5′-nucleotidase) [26]. Extracellular adenosine exerts broad immunosuppressive effects. CD39 is expressed by various immune cells and non-immune cells such as endothelial cells and fibroblasts, and by some tumor cells and intra-tumoral immune cells. In the tumor microenvironment, while ATP released by stressed or dying cells provides inflammatory signals promoting effective innate and adaptive immune responses, the hydrolysis of eATP into extracellular adenosine limits immune responses [27].

We also observed that patients whose NLR increases during ICIs treatment have poor survival compared to patients whose NLR is unchanged, and we found that a high NLR_pos_t/NLR_baseline_ is correlated with APNLR and WNT5A. The Apelin/APLNR system is increased in some cancers, is involved in tumor microenvironment reshaping and modulates tumor immune response [28].

## Conclusion

In conclusion, our results suggest a rationale for a negative prognostic significance of NLR in metastatic melanoma. It may be speculated that a high NLR results from an imbalance of circulating cells, with an increased proportion of neutrophils versus lymphocytes, but also of N2 neutrophils versus N1. The adenosine pathway seems to have a pivotal role in this altered modulation of an immune response.

### Supplementary Information

**Additional file 1: Table S1.** Toxicities other than skin type, in patients with low and high NLR. **Table S2.** Classification accuracy through the selected latent score. **Table S3.** Activity of genes in the signature. **Figure S1.** Identification of the optimal cut-point to define the subgroups of low and high NLR. **Figure S2**. Selection of best cut-point for NLR_post_/NLR_baseline_. **Figure S3.** Optimal number of components of the gene signature for baseline NLR. **Figure S4.** Variable selection by cut-off. **Figure S5.** Latent score estimation by principal component analysis, for low NLR. **Figure S6.** Latent score estimation by principal component analysis, for high NLR. **Figure S7.** Association of gene expression at baseline with response to treatment. **Figure S8.** Optimal number of components for NLR_post_/NLR_baseline_ gene signature. **Figure S9.** Latent score estimation by principal component analysis, for NLR_post_/NLR_baseline_.

## Data Availability

https://doi.org/10.5281/zenodo.7924818.

## Abbreviations

AJCC: American Joint Committee on Cancer
ANC: Absolute neutrophil count
AUC: Area under curve
CR: Complete response
DCR: Disease control rate
ERCC: External RNA Controls Consortium
ICI: Immune checkpoint inhibitor
IQR: Interquartile ranges
LDH: Lactate dehydrogenase
NLR: Neutrophil**-**to**-**lymphocyte ratio
ORR: Objective response rate
OS: Overall survival
PBMC: Peripheral blood mononuclear cells
PCA: Principal component analysis
PD: Progressive disease
PFS: Progression**-**free survival
PR: Partial response
ROC: Receiver operating characteristic
SD: Stable disease
