# Supplementary material for: CD39 and LDHA affects the prognostic role of NLR in metastatic melanoma patients treated with immunotherapy
Source: J Transl Med. 2023 Sep 8;21:610. doi: 10.1186/s12967-023-04419-6 (PMC10492378; doi:10.1186/s12967-023-04419-6)
Supplement: Supplementary file 1 — Additional file 1: Table S1. Toxicities other than skin type, in patients with low and high NLR. Table S2. Classification accuracy through the selected latent score. Table S3. Activity of genes in the signature. Figure S1. Identification of the optimal cut-point to define the subgroups of low and high NLR. Figure S2. Selection of best cut-point for NLRpost/NLRbaseline. Figure S3. Optimal number of components of the gene signature for baseline NLR. Figure S4. Variable selection by cut-off. Figure S5. Latent score estimation by principal component analysis, for low NLR. Figure S6. Latent score estimation by principal component analysis, for high NLR. Figure S7. Association of gene expression at baseline with response to treatment. Figure S8. Optimal number of components for NLRpost/NLRbaseline gene signature. Figure S9. Latent score estimation by principal component analysis, for NLRpost/NLRbaseline. [file 12967_2023_4419_MOESM1_ESM.docx]

**Additional file table 1.** Toxicities other than skin type, in patients with low and high NLR

| **Toxicity** | **Low NLR**, N = 66^1^ | **High NLR**, N = 12^1^ | **p-value**^2^ |
| --- | --- | --- | --- |
| Arthralgia | 20.0 (30.3%) | 1.0 (8.3%) | 0.27 |
| Astenia | 22.0 (33.3%) | 1.0 (8.3%) | 0.16 |
| Colitis | 12.0 (18.4%) | 1.0 (8.3%) | 0.68 |
| CPK | 1.0 (1.5%) | 0.0 (0.0%) | 1.00 |
| Diabetes | 3.0 (4.5%) | 0.0 (0.0%) | 1.00 |
| Dyspnea | 3.0 (4.5%) | 0.0 (0.0%) | 1.00 |
| Fatigue | 14.0 (21.2%) | 2.0 (16.6%) | 1.00 |
| Fever | 3.0 (4.5%) | 0.0 (0.0%) | 1.00 |
| Headache | 3.0 (4.5%) | 0.0 (0.0%) | 1.00 |
| Hyperlipasemia | 6.0 (9.0%) | 0.0 (0.0%) | 0.58 |
| Hyperthyroidism | 3.0 (4.5%) | 0.0 (0.0%) | 1.00 |
| Hypertransaminasemia | 5.0 (7.5%) | 2.0 (16.6%) | 0.27 |
| Hypothyroidism | 9.0 (13.6%) | 0.0 (0.0%) | 0.34 |
| Nausea | 9.0 (13.6%) | 0.0 (0.0%) | 0.34 |
| Pancreatitis | 7.0 (10.6%) | 1.0 (8.3%) | 1.00 |
| Skin | 29.0 (43.9%) | 1.0 (8.3%) | **0.04** |
| Stipsi | 1.0 (1.5%) | 0.0 (0.0%) | 1.00 |
| Vomit | 2.0 (3.0%) | 0.0 (0.0%) | 1.00 |
| ^1^Median (IQR) or Frequency (%) | | | |
| ^2^Fisher's exact test | | | |

**Additional file Table 2.** Classification accuracy through the selected latent score

| **Estimated** | **Original partition** | |
| --- | --- | --- |
|  | **NLR low** | **NLR high** |
| Predicted low | 64 | 1 |
| Predicted high | 2 | 11 |
| Total | 66 | 12 |
| **Index** | **Estimate** | **95% CI** |
| Sensitivity | 0.970 | 0.895–0.996 |
| Specificity | 0.912 | 0.615–0.998 |
| LR+ | 11.636 | 1.781–76.031 |
| LR- | 0.033 | 0.008–0.131 |

**Additional file Table 3.** Activity of genes in the signature

| **Gene** | **Protein full name** | **Activity** | **Taxonomy** |
| --- | --- | --- | --- |
| **CD3E** | T-cell surface glycoprotein CD3 epsilon chain | Part of the TCR-CD3 complex present on T-lymphocyte cell surface that plays an essential role in adaptive immune response. When antigen presenting cells (APCs) activate T-cell receptor (TCR), TCR-mediated signals are transmitted across the cell membrane by the CD3 chains CD3D, CD3E, CD3G and CD3Z. All CD3 chains contain immunoreceptor tyrosine-based activation motifs (ITAMs) in their cytoplasmic domain. In addition of this role of signal transduction in T-cell activation, CD3E plays an essential role in correct T-cell development. (PubMed:10384095, PubMed:26507128). | [CD3E - T-cell surface glycoprotein CD3 epsilon chain - Homo sapiens (Human) \| UniProtKB \| UniProt](https://www.uniprot.org/uniprotkb/P07766/entry) |
| **SH2D1A** | SH2 domain-containing protein 1A | Cytoplasmic adapter regulating receptors of the signaling lymphocytic activation molecule (SLAM) family such as SLAMF1, CD244, LY9, CD84, SLAMF6 and SLAMF7. In SLAM signaling seems to cooperate with SH2D1B/EAT-2. Positively regulates CD244/2B4- and CD84-mediated natural killer (NK) cell functions. Can also promote CD48-, SLAMF6 -, LY9-, and SLAMF7-mediated NK cell activation. In the context of NK cell-mediated cytotoxicity enhances conjugate formation with target cells (By similarity). May also regulate the activity of the neurotrophin receptors NTRK1, NTRK2 and NTRK3 | [SH2D1A - SH2 domain-containing protein 1A - Homo sapiens (Human) \| UniProtKB \| UniProt](https://www.uniprot.org/uniprotkb/O60880/entry) |
| **ZAP70** | Tyrosine-protein kinase ZAP-70 | Tyrosine kinase that plays an essential role in regulation of the adaptive immune response. Regulates motility, adhesion and cytokine expression of mature T-cells, as well as thymocyte development. Contributes also to the development and activation of primary B-lymphocytes. When antigen presenting cells (APC) activate T-cell receptor (TCR), a serie of phosphorylations lead to the recruitment of ZAP70 to the doubly phosphorylated TCR component CD247/CD3Z through ITAM motif at the plasma membrane. This recruitment serves to localization to the stimulated TCR and to relieve its autoinhibited conformation. Release of ZAP70 active conformation is further stabilized by phosphorylation mediated by LCK. Subsequently, ZAP70 phosphorylates at least 2 essential adapter proteins: LAT and LCP2. In turn, a large number of signaling molecules are recruited and ultimately lead to lymphokine production, T-cell proliferation and differentiation. Furthermore, ZAP70 controls cytoskeleton modifications, adhesion and mobility of T-lymphocytes, thus ensuring correct delivery of effectors to the APC. | [ZAP70 - Tyrosine-protein kinase ZAP-70 - Homo sapiens (Human) \| UniProtKB \| UniProt](https://www.uniprot.org/uniprotkb/P43403/entry) |
| **CD45RA** | Receptor-type tyrosine-protein phosphatase C | Protein tyrosine-protein phosphatase required for T-cell activation through the antigen receptor. Acts as a positive regulator of T-cell coactivation upon binding to DPP4. The first PTPase domain has enzymatic activity, while the second one seems to affect the substrate specificity of the first one. Upon T-cell activation, recruits and dephosphorylates SKAP1 and FYN. Dephosphorylates LYN, and thereby modulates LYN activity (By similarity). | [PTPRC - Receptor-type tyrosine-protein phosphatase C - Homo sapiens (Human) \| UniProtKB \| UniProt](https://www.uniprot.org/uniprotkb/P08575/entry) |
| **CCNA1** | Cyclin-A1 | May be involved in the control of the cell cycle at the G1/S (start) and G2/M (mitosis) transitions. May primarily function in the control of the germline meiotic cell cycle and additionally in the control of mitotic cell cycle in some somatic cells. | [CCNA1 - Cyclin-A1 - Homo sapiens (Human) \| UniProtKB \| UniProt](https://www.uniprot.org/uniprotkb/P78396/entry) |
| **LDHA** | L-lactate dehydrogenase A chain | Interconverts simultaneously and stereospecifically pyruvate and lactate with concomitant interconversion of NADH and NAD+.B | [ldha - L-lactate dehydrogenase A chain - Squalus acanthias (Spiny dogfish) \| UniProtKB \| UniProt](https://www.uniprot.org/uniprotkb/P00341/entry) |
| **IL18R1** | Interleukin-18 receptor 1 | Within the IL18 receptor complex, responsible for the binding of the pro-inflammatory cytokine IL18, but not IL1A nor IL1B (PubMed:8626725, PubMed:14528293, PubMed:25261253, PubMed:25500532). Involved in IL18-mediated IFNG synthesis from T-helper 1 (Th1) cells (PubMed:10653850). | [IL18R1 - Interleukin-18 receptor 1 - Homo sapiens (Human) \| UniProtKB \| UniProt](https://www.uniprot.org/uniprotkb/Q13478/entry) |

**Additional file Figure 1.** Identification of the optimal cut-point to define the subgroups of low and high NLR

**Additional file Figure 2**. Selection of best cut-point for NLR_post_/NLR_baseline_

**Additional file Figure 3.** Optimal number of components of the gene signature for baseline NLR

**Additional file Figure 4**. Variable selection by cut-off

**Additional file Figure 5.** Latent score estimation by principal component analysis, for low NLR

**Additional file Figure 6**. Latent score estimation by principal component analysis, for high NLR

**Supplememntary Figure 7.** Association of gene expression at baseline with response to treatment

**Additional file Figure 8**. Optimal number of components for NLR_post_/NLR_baseline_ gene signature

**Additional file Figure 9**. Latent score estimation by principal component analysis, for NLR_post_/NLR_baseline_
